# Supplementary material for: Associations between Respiratory Health Outcomes and Coal Mine Fire PM2.5 Smoke Exposure: A Cross-Sectional Study
Source: Int J Environ Res Public Health. 2019 Nov 2;16(21):4262. doi: 10.3390/ijerph16214262 (PMC6862448; doi:10.3390/ijerph16214262)
Supplement: Supplementary file 1 [file ijerph-16-04262-s001.pdf]

Supplementary Materials

Supplementary Table S1. Imputed observations for self-reported survey variables.

Predictor Variable	Complete	No. Imputed	% Imputed	Total
Age	3090	6	0.19%	3096
Employment	3057	39	1.26%	3096
Education	3055	41	1.32%	3096
Smoking	3063	33	1.07%	3096
Male	3094	2	0.06%	3096
Married	3078	18	0.58%	3096
Wheeze	3089	7	0.23%	3096
Chest tightness	3089	7	0.23%	3096
Nocturnal shortness of breath	3089	7	0.23%	3096
Resting shortness of breath	3088	8	0.26%	3096
Current nasal symptoms	3088	8	0.26%	3096
Cough	3088	8	0.26%	3096
Phlegm	3088	8	0.26%	3096
Asthma pre 2014	3079	17	0.55%	3096
COPD pre 2014	3086	10	0.32%	3096
Asthma since 2014	3079	17	0.55%	3096
COPD since 2014	3086	10	0.32%	3096

Supplementary Table S2. Whole Population - Multivariate model results for associations between respiratory outcomes and mine fire-related $PM_{2.5}$ concentrations.

	**Mean 24-h exposure model (10 µg/m³)**		**Peak 12-h exposure model (100 µg/m³)**	
**Outcomes**	**Adj OR (95% CI)**	**p-value**	**Adj OR (95% CI)**	**p-value**
Current wheeze	1.04 (0.96,1.12)	0.391	1.06 (1.02,1.11)	0.004
Chest tightness	1.04 (0.95,1.12)	0.403	1.03 (0.99,1.08)	0.129
Nocturnal shortness of breath	1.01 (0.92,1.10)	0.833	1.02 (0.98,1.07)	0.332
Resting shortness of breath	1.02 (0.93,1.11)	0.704	1.03 (0.98,1.08)	0.237
Current nasal symptoms	1.03 (0.95,1.11)	0.481	1.02 (0.98,1.06)	0.288
Chronic cough	1.13 (1.03,1.23)	0.007	1.07 (1.02,1.12)	0.004
Chronic Phlegm	1.10 (1.00,1.20)	0.052	1.04 (0.99,1.09)	0.153
Asthma since 2014	1.08 (0.85,1.37)	0.530	1.08 (0.93,1.25)	0.341
COPD since 2014	0.84 (0.64,1.10)	0.197	0.96 (0.83,1.11)	0.573

Supplementary Table S3. Sex stratified results for males - Multivariate model results for associations between respiratory outcomes and mine fire-related $PM_{2.5}$ concentrations.

	Mean 24-h exposure model (10 μg/m³)		Peak 12-h exposure model (100 μg/m³)	
**Outcomes**	**Adj OR (95% CI)**	**p-value**	**Adj OR (95% CI)**	**p-value**
Current wheeze	1.11 (0.97,1.26)	0.120	1.10 (1.03,1.17)	0.004
Chest tightness	1.08 (0.96,1.22)	0.216	1.04 (0.98,1.11)	0.212
Nocturnal shortness of breath	1.11 (0.98,1.26)	0.112	1.07 (1.00,1.14)	0.067
Resting shortness of breath	1.08 (0.94,1.24)	0.289	1.06 (0.98,1.14)	0.128
Current nasal symptoms	1.03 (0.91,1.15)	0.660	1.02 (0.96,1.08)	0.573
Chronic cough	1.20 (1.05,1.37)	0.007	1.07 (1.00,1.14)	0.050
Chronic Phlegm	1.13 (0.99,1.28)	0.064	1.04 (0.97,1.11)	0.285
Asthma since 2014	1.58 (1.10,2.29)	0.014	1.43 (1.14,1.78)	0.002
COPD since 2014	0.76 (0.51,1.14)	0.182	0.90 (0.72,1.12)	0.342

Supplementary Table S4. Sex stratified results for females - Multivariate model results for associations between respiratory outcomes and mine fire-related $PM_{2.5}$ concentrations.

	Mean 24-h exposure model (10 μg/m³)		Peak 12-h exposure model (100 μg/m³)	
**Outcomes**	**Adj OR (95% CI)**	**p-value**	**Adj OR (95% CI)**	**p-value**
Current wheeze	0.97 (0.88,1.06)	0.497	1.03 (0.97,1.08)	0.363
Chest tightness	1.00 (0.90,1.10)	0.937	1.03 (0.97,1.09)	0.374
Nocturnal shortness of breath	0.93 (0.82,1.04)	0.199	0.98 (0.92,1.05)	0.609
Resting shortness of breath	0.97 (0.87,1.09)	0.663	1.00 (0.94,1.07)	0.882
Current nasal symptoms	1.03 (0.94,1.13)	0.472	1.03 (0.98,1.08)	0.305
Chronic cough	1.05 (0.94,1.17)	0.368	1.06 (1.00,1.12)	0.051
Chronic Phlegm	1.05 (0.92,1.20)	0.461	1.03 (0.96,1.11)	0.407
Asthma since 2014	0.83 (0.63,1.09)	0.175	0.84 (0.70,1.01)	0.060
COPD since 2014	0.93 (0.66,1.30)	0.652	1.01 (0.85,1.19)	0.944

Supplementary Table S5. Age stratified results for those aged 18-64 years - Multivariate model results for associations between respiratory outcomes and mine fire-related $PM_{2.5}$ concentrations.

	Mean 24-h exposure model (10 µg/m³)		Peak 12-h exposure model (100 µg/m³)	
**Outcomes**	**Adj OR (95% CI)**	**p-value**	**Adj OR (95% CI)**	**p-value**
Current wheeze	1.08 (0.98,1.20)	0.131	1.07 (1.02,1.13)	0.006
Chest tightness	1.06 (0.96,1.16)	0.276	1.04 (0.98,1.09)	0.171
Nocturnal shortness of breath	1.04 (0.93,1.15)	0.530	1.03 (0.97,1.09)	0.350
Resting shortness of breath	1.06 (0.95,1.18)	0.272	1.04 (0.98,1.10)	0.196
Current nasal symptoms	1.05 (0.95,1.16)	0.303	1.03 (0.98,1.09)	0.211
Chronic cough	1.17 (1.04,1.30)	0.008	1.07 (1.02,1.14)	0.013
Chronic Phlegm	1.14 (1.02,1.28)	0.023	1.04 (0.98,1.10)	0.203
Asthma since 2014	1.11 (0.84,1.47)	0.464	1.10 (0.93,1.31)	0.279
COPD since 2014	0.91 (0.64,1.28)	0.572	0.94 (0.76,1.16)	0.586

Supplementary Table S6. Age stratified results for those aged 65 years and over - Multivariate model results for associations between respiratory outcomes and mine fire-related $PM_{2.5}$ concentrations.

	Mean 24-h exposure model (10 µg/m³)		Peak 12-h exposure model (100 µg/m³)	
**Outcomes**	**Adj OR (95% CI)**	**p-value**	**Adj OR (95% CI)**	**p-value**
Current wheeze	0.94 (0.83,1.07)	0.377	1.02 (0.95,1.10)	0.504
Chest tightness	0.97 (0.84,1.11)	0.640	1.01 (0.94,1.09)	0.794
Nocturnal shortness of breath	0.92 (0.81,1.06)	0.240	0.99 (0.92,1.06)	0.753
Resting shortness of breath	0.90 (0.78,1.05)	0.181	0.99 (0.91,1.07)	0.797
Current nasal symptoms	0.97 (0.88,1.08)	0.601	0.99 (0.93,1.05)	0.626
Chronic cough	1.04 (0.92,1.18)	0.510	1.04 (0.97,1.12)	0.241
Chronic Phlegm	0.97 (0.83,1.14)	0.699	1.01 (0.93,1.11)	0.769
Asthma since 2014	1.04 (0.72,1.50)	0.846	0.99 (0.81,1.22)	0.939
COPD since 2014	0.79 (0.54,1.16)	0.227	0.98 (0.82,1.18)	0.829

Supplementary Table S7. Whole Population - Multivariate model results for associations between respiratory outcomes and a) Home building materials and b) Roofing materials.

		Mean 24-h exposure model (10 μg/m³)		Peak 12-h exposure model (100 μg/m³)	
**Outcomes**	**Material Type**	**Adj OR (95% CI)**	**p-value**	**Adj OR (95% CI)**	**p-value**
Current wheeze	Home main material - brick/concrete	0.74 (0.61,0.90)	0.002	0.75 (0.62,0.91)	0.004
	Roof main material - tin/iron	0.93 (0.76,1.14)	0.474	0.94 (0.76,1.15)	0.523
Chest tightness	Home main material - brick/concrete	0.83 (0.67,1.02)	0.082	0.84 (0.68,1.03)	0.089
	Roof main material - tin/iron	1.09 (0.89,1.35)	0.396	1.10 (0.89,1.35)	0.372
Nocturnal shortness of breath	Home main material - brick/concrete	0.79 (0.63,0.99)	0.040	0.80 (0.63,1.00)	0.047
	Roof main material - tin/iron	0.95 (0.76,1.20)	0.684	0.96 (0.76,1.20)	0.698
Resting shortness of breath	Home main material - brick/concrete	0.74 (0.59,0.93)	0.010	0.75 (0.59,0.94)	0.012
	Roof main material - tin/iron	1.00 (0.78,1.27)	0.979	1.00 (0.79,1.27)	1.000
Current nasal symptoms	Home main material - brick/concrete	0.88 (0.73,1.05)	0.156	0.88 (0.73,1.05)	0.161
	Roof main material - tin/iron	1.25 (1.04,1.50)	0.019	1.25 (1.04,1.51)	0.017
Chronic cough	Home main material - brick/concrete	0.79 (0.65,0.97)	0.021	0.79 (0.65,0.96)	0.016
	Roof main material - tin/iron	1.12 (0.91,1.38)	0.284	1.13 (0.92,1.39)	0.236
Chronic Phlegm	Home main material - brick/concrete	0.87 (0.70,1.08)	0.201	0.86 (0.69,1.06)	0.163
	Roof main material - tin/iron	1.26 (1.01,1.57)	0.038	1.27 (1.02,1.59)	0.032
Asthma since 2014	Home main material - brick/concrete	1.19 (0.58,2.41)	0.635	1.20 (0.59,2.42)	0.612
	Roof main material - tin/iron	1.12 (0.53,2.37)	0.766	1.13 (0.53,2.40)	0.748
COPD since 2014	Home main material - brick/concrete	1.16 (0.65,2.06)	0.613	1.19 (0.67,2.12)	0.555
	Roof main material - tin/iron	1.27 (0.71,2.26)	0.423	1.23 (0.69,2.20)	0.488

Supplementary Table S8. Multivariate analysis results when a two-way interaction was fitted between mine fire $PM_{2.5}$ concentrations and housing material.

Outcomes		Mean 24-h exposure model (10 µg/m³)		Peak 12-h exposure model (100 µg/m³)	
		Adj OR (95% CI)	p-value	Adj OR (95% CI)	p-value
Current wheeze	Main effect	1.10 (0.97,1.25)	0.132	1.09 (1.02,1.16)	0.011
	Interaction	0.90 (0.76,1.05)	0.185	0.96 (0.88,1.04)	0.338
Chest tightness	Main effect	1.10 (0.97,1.24)	0.126	1.06 (0.99,1.14)	0.076
	Interaction	0.90 (0.76,1.06)	0.208	0.95 (0.87,1.04)	0.293
Nocturnal shortness of breath	Main effect	1.13 (0.99,1.28)	0.062	1.06 (0.99,1.14)	0.114
	Interaction	0.81 (0.68,0.97)	0.025	0.94 (0.85,1.03)	0.202
Resting shortness of breath	Main effect	1.15 (1.01,1.30)	0.036	1.08 (1.00,1.16)	0.039
	Interaction	0.80 (0.66,0.96)	0.015	0.92 (0.83,1.01)	0.077
Current nasal symptoms	Main effect	1.07 (0.95,1.21)	0.253	1.04 (0.98,1.12)	0.203
	Interaction	0.93 (0.80,1.08)	0.329	0.96 (0.89,1.05)	0.383
Chronic cough	Main effect	1.10 (0.96,1.26)	0.178	1.07 (1.00,1.15)	0.056
	Interaction	1.05 (0.88,1.25)	0.605	0.99 (0.91,1.09)	0.873
Chronic Phlegm	Main effect	1.09 (0.96,1.24)	0.166	1.04 (0.97,1.11)	0.258
	Interaction	1.00 (0.84,1.21)	0.964	0.99 (0.90,1.10)	0.908
Asthma since 2014	Main effect	0.97 (0.71,1.34)	0.858	1.05 (0.79,1.39)	0.753
	Interaction	1.17 (0.77,1.79)	0.465	1.04 (0.76,1.44)	0.791
COPD since 2014	Main effect	0.92 (0.63,1.32)	0.641	0.97 (0.78,1.19)	0.763
	Interaction	0.86 (0.51,1.46)	0.579	0.98 (0.74,1.31)	0.916

Note: The reported Interaction result reflects the association between respiratory outcomes and the two-way interaction variable ($PM_{2.5}$ and housing material).

The reported Main effect result reflects the association between respiratory outcomes and mine fire $PM_{2.5}$ concentrations for participants with non-brick/concrete building materials.

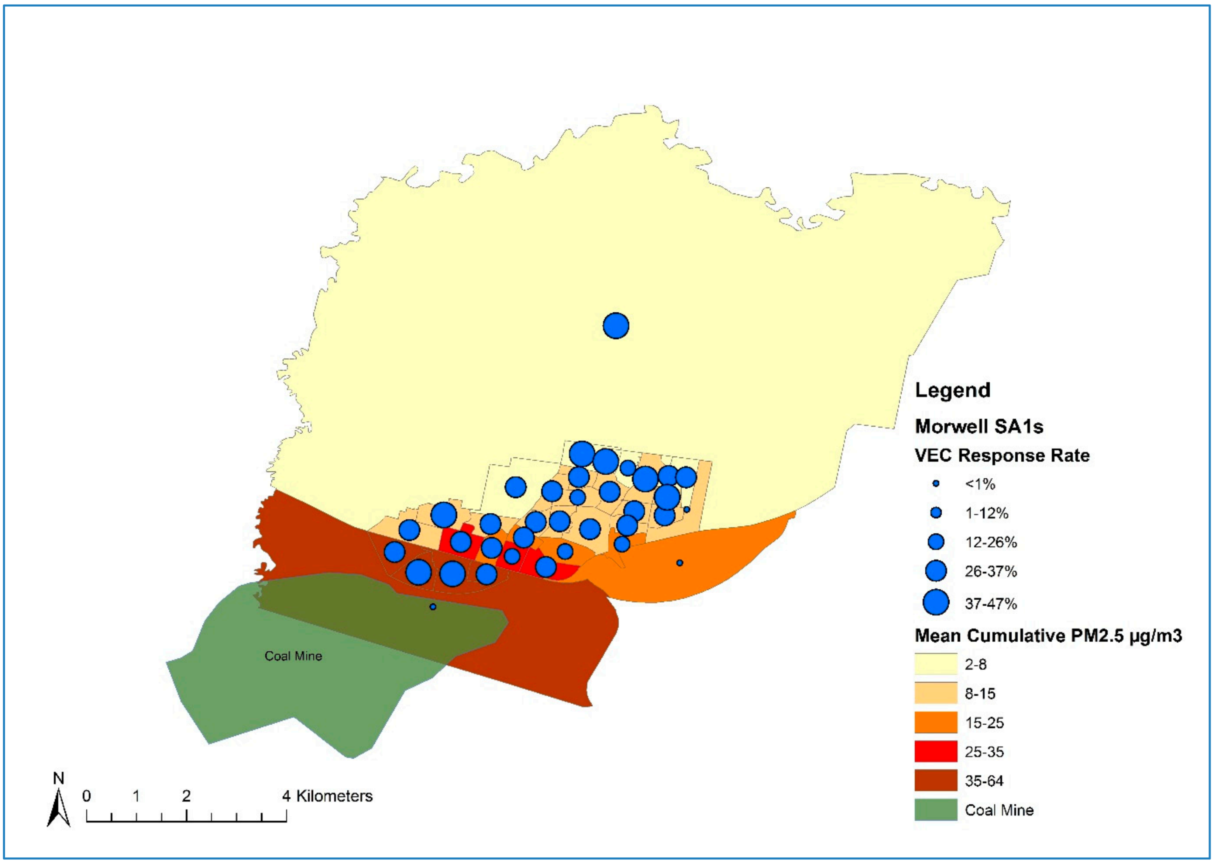

Supplementary Figure S1. Morwell Adult Survey VEC participation rates by SA1, mapped on mean cumulative 24-h mine fire-related $PM_{2.5}$ concentrations, for the period 9 February – 31 March 2014.

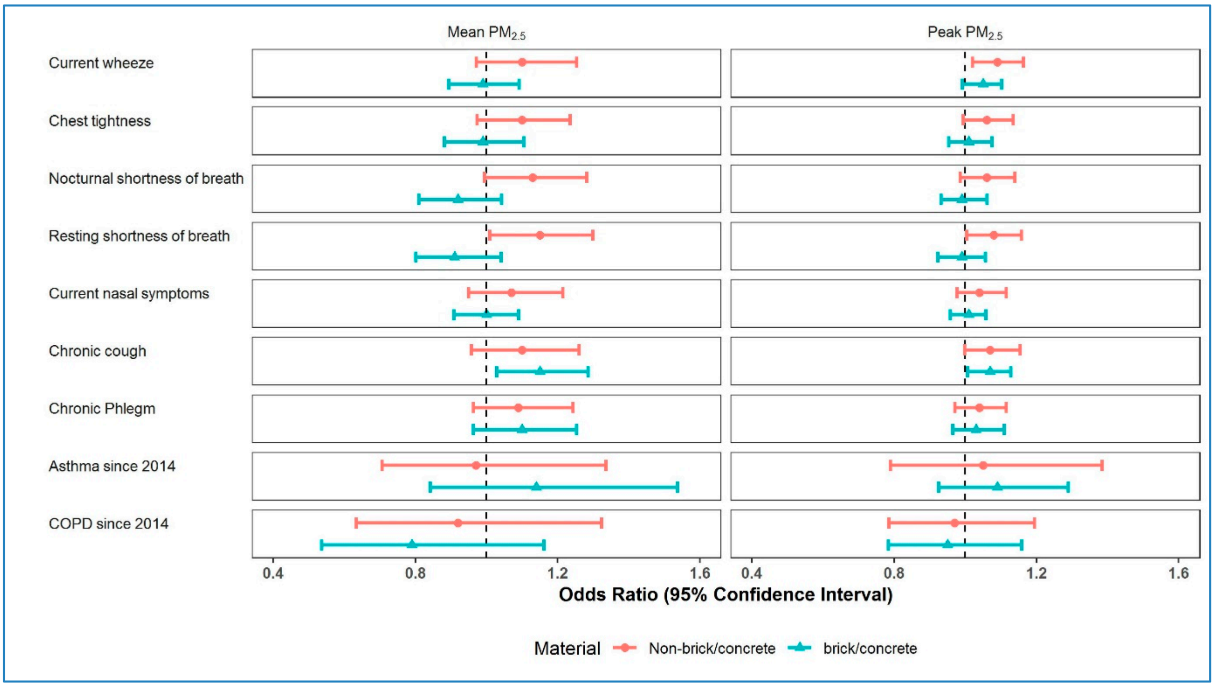

Supplementary Figure S2. Multivariate analysis results of the associations between mine fire-related $PM_{2.5}$ concentrations and respiratory outcomes stratified by housing material, when a variable is included for the interaction between $PM_{2.5}$ concentrations and housing building materials.
